# Did the dislocation risk after primary total hip arthroplasty decrease over time? A meta-analysis across six decades

**DOI:** 10.1007/s00402-022-04678-w

**Published:** 2022-11-10

**Authors:** J. H. J. van Erp, M. F. T. Hüsken, M. D. Filipe, T. E. Snijders, M. C. Kruyt, A. de Gast, T. P. C. Schlösser

**Affiliations:** 1 Clinical Orthopedic Research Center–mN, Diakonessenhuis, Professor Lorentzlaan 76, 3707 HL Zeist, The Netherlands; 2 Department of Orthopedic Surgery, Diakonessenhuis, Utrecht, The Netherlands; 3 Department of Orthopaedic Surgery, University Medical Center Utrecht, Utrecht, The Netherlands; 4 Department of Surgery, University Medical Center Utrecht, Utrecht, The Netherlands; 5 Department of Developmental Bioengineering, Twente University, Enschede, The Netherlands

**Keywords:** Total hip artroplasty, THA, Dislocation, Instabilty, RCT, Registry

## Abstract

**Background:**

While continuous optimization is attempted to decrease the incidence of dislocation after total hip arthroplasty (THA), dislocation remains a major complication. This meta-analysis aims to analyze the evolution of the dislocation risk after primary THA over the decades and to evaluate its potential publication bias.

**Patients and methods:**

A systematic search was performed according to the PRISMA guidelines for this meta-analysis in the literature published between 1962 and 2020. MEDLINE, Cochrane and Embase databases were searched for studies reporting the dislocation risk and length of follow-up. Studies that reported on revision rates only and did not mention separate dislocations were excluded. All study designs were eligible. Study quality was assessed by existing quality assessment tools adjusted for arthroplasty research. Overall risk and yearly dislocation rates were calculated and related to historical time frame, study design, sample size and length of follow-up.

**Results:**

In total, 174 studies were included with an overall moderate quality. In total there were 85.209 dislocations reported in 5.030.293 THAs, showing an overall dislocation risk of 1.7%, with a median follow-up of 24 months. The overall dislocation risk classified per decade decreased from 3.7% in 1960–1970 to 0.7% in 2010–2020. The yearly dislocation rate decreased from 1.8 to 0.7% within these same decades. There was no significant correlation between the reported dislocation risk and the duration of follow-up (*p* = 0.903) or sample size (*p* = 0.755). The reported dislocation risk was higher in articles with registry data compared to other study designs (*p* = 0.021).

**Conclusion:**

The dislocation risk in THA has been decreasing over the past decades to 0.7%. Non-selective registry studies reported a higher dislocation risk compared to studies with selective cohorts and RCTs. This indicates that the actual dislocation risk is higher than often reported and ‘real-world data’ are reflected better in large-scale cohorts and registries.

**Supplementary Information:**

The online version contains supplementary material available at 10.1007/s00402-022-04678-w.

## Introduction

Total hip arthroplasty (THA) is a common orthopedic procedure that has been declared the most successful operation of the twentieth century, being very effective in relieving pain and improving hip function [1]. Due to aging population, the number of primary, and revision THAs is increasing [2]. Surgical techniques and design of the prostheses have been improved since its introduction, in order to optimize clinical outcomes and component survival. A clear example is that various studies demonstrated that larger head size results in a lower risk of dislocation [3–8]. However, dislocation remains a major and relatively frequent complication. The majority of the THA dislocations occur in the early postoperative period, but even after years dislocation remains an issue. These late dislocations are mostly a result of wear, precarious movement or an altered spinopelvic mobility [3, 9–13].

At its introduction in 1962, the Charnley ‘low friction’ arthroplasty had a dislocation risk of 4.8% [14]. In 2010, a very large and long-term Medicare cohort study showed a similar dislocation risk of 4.8%, suggesting that the dislocation risk may have not reduced since 1961 [15]. However, studies introducing novel or modified techniques often suggest that these result in a lower risk of dislocation. Unfortunately, those studies often represent a selective cohort and have a relatively short follow-up, retrospective design or small sample.

The aim of this meta-analysis is a thorough evaluation of dislocations rates of primary THAs over the decades and evaluation of the effect of study design, sample size and length of follow-up, hypothesizing a decrease in dislocation risk with a more accurate representation of ‘real-world data’ in large, non-selective cohort and registry studies.

## Methods

### Search strategy and selection criteria

A systematic search was conducted to collect all research reporting dislocation risk or rates after THA, according to the guidelines of the PRISMA Checklist for reporting systematic reviews and meta-analyses [16]. A search was performed using the MEDLINE, Cochrane Library and Embase databases in December 2020. The search syntax was constructed from the terms ‘total hip arthroplasty,’ ‘dislocation,’ ‘primary’ and its corresponding synonyms in singular and plural. The full search strategy can be found in supplementary data (Supplementary 1). After removal of duplicates, two authors (Author 1 and 2) independently screened articles by title and abstract. Only publications written in English and published after 1962 were considered for review. The full articles were independently screened for eligibility based on predefined inclusion and exclusion criteria. All studies reporting on the dislocation risk of primary THA, with a known duration of follow-up were included. Reviews and meta-analyses were excluded and so were animal studies, studies with < 100 THAs and studies with a follow-up < 1 month. Studies reporting on revision THA because of instability without a separate dislocation rate, resurfacing THA, metal-on-metal THA or dual mobility devices were excluded as well, except for studies reporting dislocation risk of a primary THA population as control group separately. Studies in which the majority of the patients had comorbidities which have a known influence on the dislocation rate were excluded as well, such as Parkinson’s disease, epilepsy, dementia or previous lumbar surgery [3, 4, 10, 17]. In case of multiple studies reporting on a single cohort, only the publication with the longest follow-up was included. Discordant judgments were discussed by two authors until consensus was achieved.

### Quality assessment

To assess the quality of the included studies, a checklist with criteria drawn up by Wylde et al. and Evans et al. was used [18, 19]. This checklist is based on existing quality assessment tools for prospective and retrospective studies (MINORs, Newcastle–Ottawa Quality Assessment Scale, ROBINS-I) [20, 21]. Since a relatively high number of patients fails to complete long-term follow-up after THA and several criteria are irrelevant to joint replacement, the checklist was adjusted for THA research. Studies were evaluated for consecutive inclusion, multicenter setting, follow-up of > 80% of the population and the use of multivariate analysis. The individual item ratings were reported as adequate, not adequate or not reported. Ratings of methodological quality of the included studies were conducted by one reviewer and discussed with a second in case of doubt.

### Data extraction

The following data were extracted from the included articles: author, year of publication, sample size, years at which the THAs were implanted, mean follow-up, study design, number of THAs and number of dislocated THAs during the study. Only studies which described the percentage or actual number of dislocations were included. Studies that only described a revision rate were excluded, since a considerable part of the patients experience dislocation only without need for revision [3, 22, 23]. Data on surgical techniques and patient factors were not extracted because they were not available, or not comparable in a lot of studies.

The overall dislocation risk was calculated by the total number of dislocated THAs from all studies and divided by the total number of THAs. The studies were categorized by decade, based on the year in which the first surgeries were performed. The overall dislocation risk was calculated for each decade and analyzed continuously. Yearly dislocation rates were calculated by dividing the dislocation rate by the mean follow-up time. Studies with a follow-up less than one year were excluded for these analyses. The effect of the length of follow-up and sample size on the reported dislocation risk were analyzed as a continuous parameter. In addition, we calculated the overall dislocation rate and yearly risk for the different study designs and for studies with and without ‘real-world data’ (RWD). This was defined as studies with a minimum of 1000 patients and 1-year follow-up.

### Statistical analysis

The data were assessed using R 3.6.2 (The R Foundation for Statistical Computing, Vienna, Austria). Statistical packages used were *psych, meta* and *metaphor.* All graphs were made using the *ggplot2* package [24]. Heterogeneity among studies was quantified by the I-square and tested using Cochran’s Chi-square tests. Normality was assessed by comparing mean to median and by testing for skewness and kurtosis using Shapiro test. Funnel plots were used to explore possible asymmetry or an unequal distribution of studies. Reported dislocation rates were compared using the Mann–Whitney test and the Kruskal–Wallis test, and *p* < 0.05 was considered statistically significant. Additionally, multivariate meta-regression analysis was used to determine the influence of follow-up duration on the reported dislocation risk, after adjusting for design sample size and decade.

## Results

### Search results

**Fig. 1:**
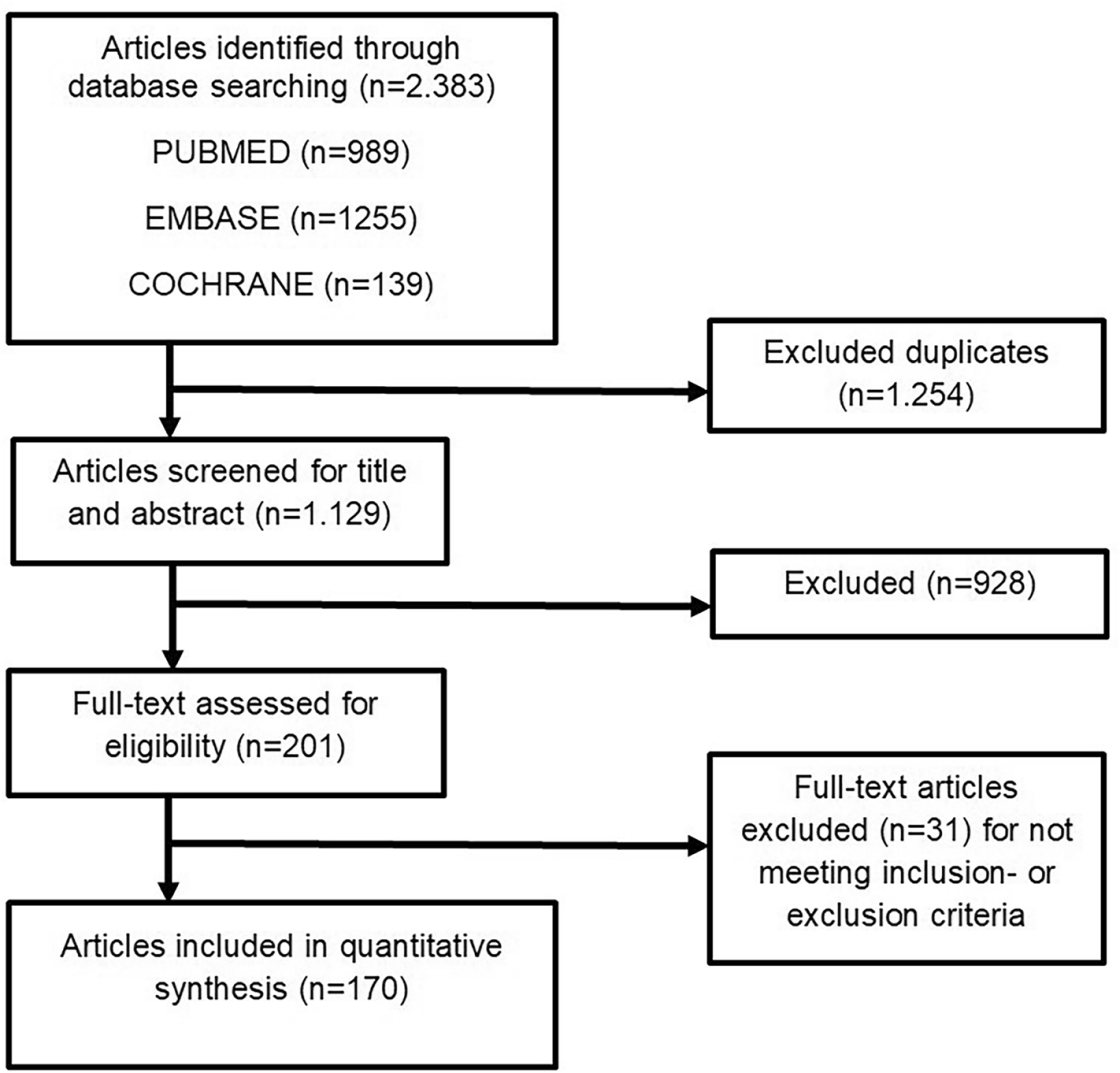
Flowchart showing the process of inclusion

Our search yielded a total of 2383 publications. After applying the in- and exclusion criteria to full-text articles, a total of 170 unique publications remained (Fig. 1)*.* Three studies included cohorts that covered more than one decade, and one study studied both a short-term and a long-term cohort. These four were included as separate series in the statistical analysis, resulting in a total of 174 THA populations. Five studies were RCTs, 32 registry studies (studies using national or regional implant/health/insurance registers), 46 prospective and 91 retrospective cohort studies  97 (45.4%) studies reported on > 1000 THAs. The mean length of follow-up was 36 months, with a median of 24 months (range 1–192).

The quality of included studies was moderate. The assessment showed that 78 (44.9%) studies were consecutive, 39 (22.9%) multicenter, 90 (52.9%) had less than 20% loss to follow-up and 53 (31.2%) used multivariable analyses. (Table 1 Supplementary). None of the overall dislocation rates and risks were normally distributed and therefore described using median and interquartile range (IQR).

**Table 1:** Baseline characteristics and total and yearly dislocation rates are shown

Study years	1970–1979	1980–1989	1990–1999	2000–2009	2010–2019	Total
Number of studies	10	18	47	73	26	174
Follow-up in months (median)	37	52.5	24	24	12	24
*n* THAs	60.86	35.702	551.511	3.698.220	684	5.030.293
*n* of dislocated THAs	2.232	828	16.3	61.139	4.708	85.207
Overall dislocation risk	3.70%	2.30%	3.00%	1.70%	0.70%	1.70%
Yearly dislocation rate*	1.80%	1.00%	1.30%	0.90%	0.70%	1.00%

*Yearly dislocation rates are based on studies with a minimum of 1-year follow-up

### Overall dislocation risk

The included studies involved a total of 5.030.293 THAs, of which 85.209 had dislocated, resulting in an overall dislocation risk of 1.7% (Table 1). The yearly dislocation rate was on average 1.0% per year of follow-up.

### Historic comparison

**Fig. 2:**
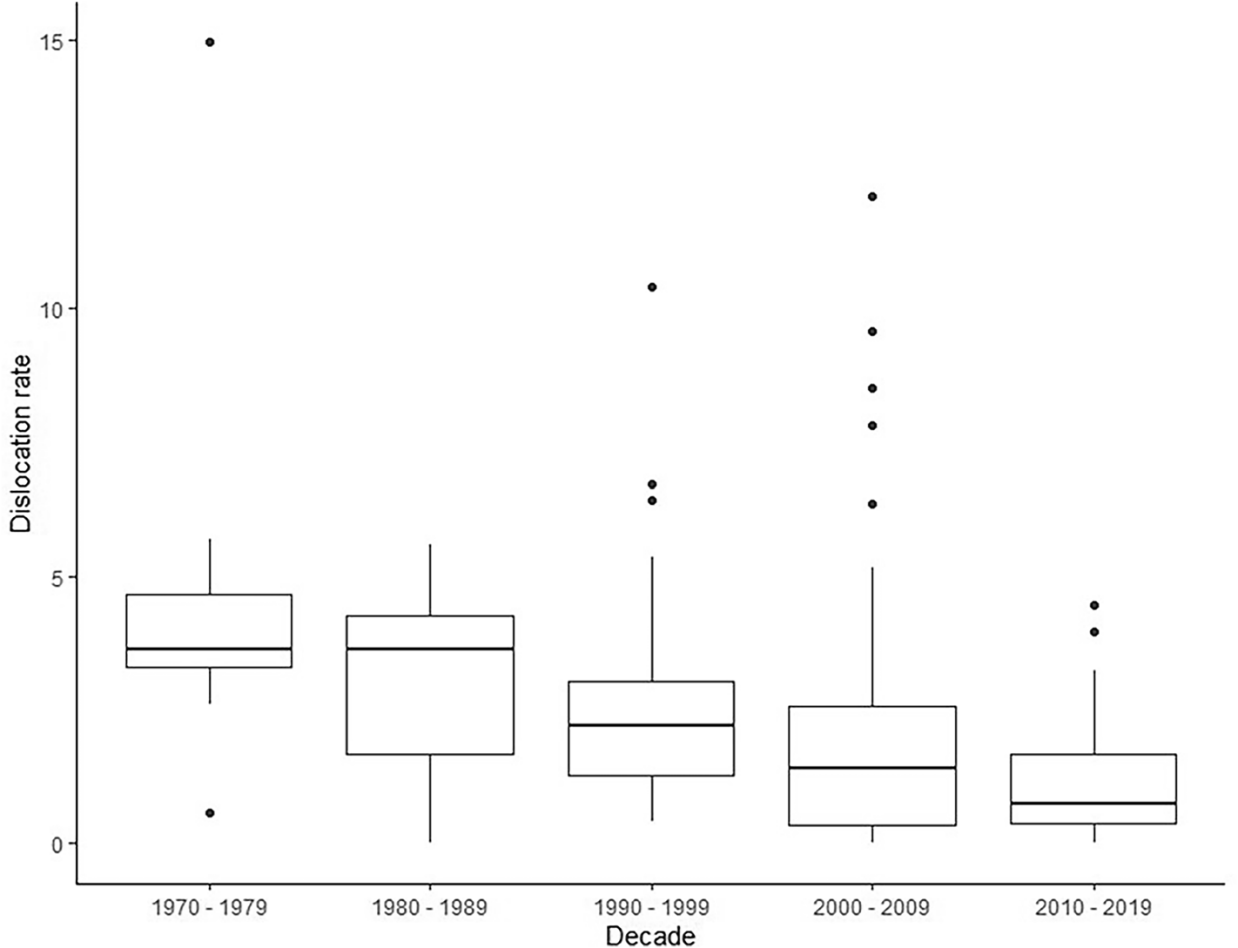
Dislocation rate (%) per decade from 1970 to 2019

A decline in reported dislocation risk was observed over the past 50 years (*p* < 0.001) (Tables 1 and 2, and Fig. 2): The overall dislocation risk declined from 3.7 to 0.7% from 1962 to 2020. The median follow-up and the yearly dislocation rate also decreased, but not significantly, from 37.1 to 12.5 months and from 1.8 to 0.7%, respectively. The greatest decline in overall dislocation risk was observed from 1970 to 1980. After adjustment for confounders (follow-up, sample size and design), each consecutive year the overall dislocation risk decreased 4.7% relatively to the previous year (estimate = − 0.047; 95% CI − 0.062 − 0.032; *p* < 0.001).

**Table 2:** Dislocation risk per decade, stratified by follow-up period

	< 1 year FU	1–2 years FU	2–5 years FU	5–10 years FU	> 10 years FU	*p* value
1970–1979	*n*: 0	*n*: 2	*n*: 4	*n*: 3	*n*: 1	0.597
	NA	3.29	4.52	3.87	NA	
		[2.28–3.30]	[2.65–8.00]	[3.24–4.00]		
1980–1989	*n*: 2	*n*: 2	*n*: 5	*n*: 9	*n*: 0	0.2404
	4.04	4.81	3.41	2.78	NA	
	[3.83–4.24]	[4.43–5.20]	[1.09–3.74]	[1.59–4.35]		
1990–1999	*n*: 12	*n*: 9	*n*: 14	*n*: 9	*n*: 3	0.783
	1.8	2.32	2.22	2.4	1.72	
	[1.20–2.91]	[1.83–4.00]	[1.16–2.51]	[1.16–3.27]	[1.52–2.17]	
2000–2009	*n*: 12	*n*: 21	*n*: 28	*n*: 9	*n*: 3	0.117
	1.45	1.03	1.66	0.55	8.49	
	[0.31–3.41]	[0.47–2.19]	[0.73–2.53]	[0.00–1.11]	[4.33–10.29]	
2010–2019	*n*: 9	*n*: 8	*n*: 8	*n*: 1	*n*: 0	0.453
	0.63	0.71	1.31	NA	NA	
	[0.46–1.37]	[0.33–1.62]	[0.44–2.61]			
Total	*N* = 35	*N* = 42	*N* = 59	*N* = 30	*N* = 8	0.648
	1.43	1.61	2.04	1.73	2.58	
	[0.60–2.97]	[0.57–3.20]	[0.91–3.23]	[0.81–3.81]	[1.52–5.75]	

*FU* follow-up, *RWD* real-world data, as defined as a minimum of 1000 patients and 1 year FU

*Yearly dislocation rates are based on data from studies with a minimum of 1-year follow-up

### Risk of publication bias in reporting of THA dislocations

**Fig. 3:**
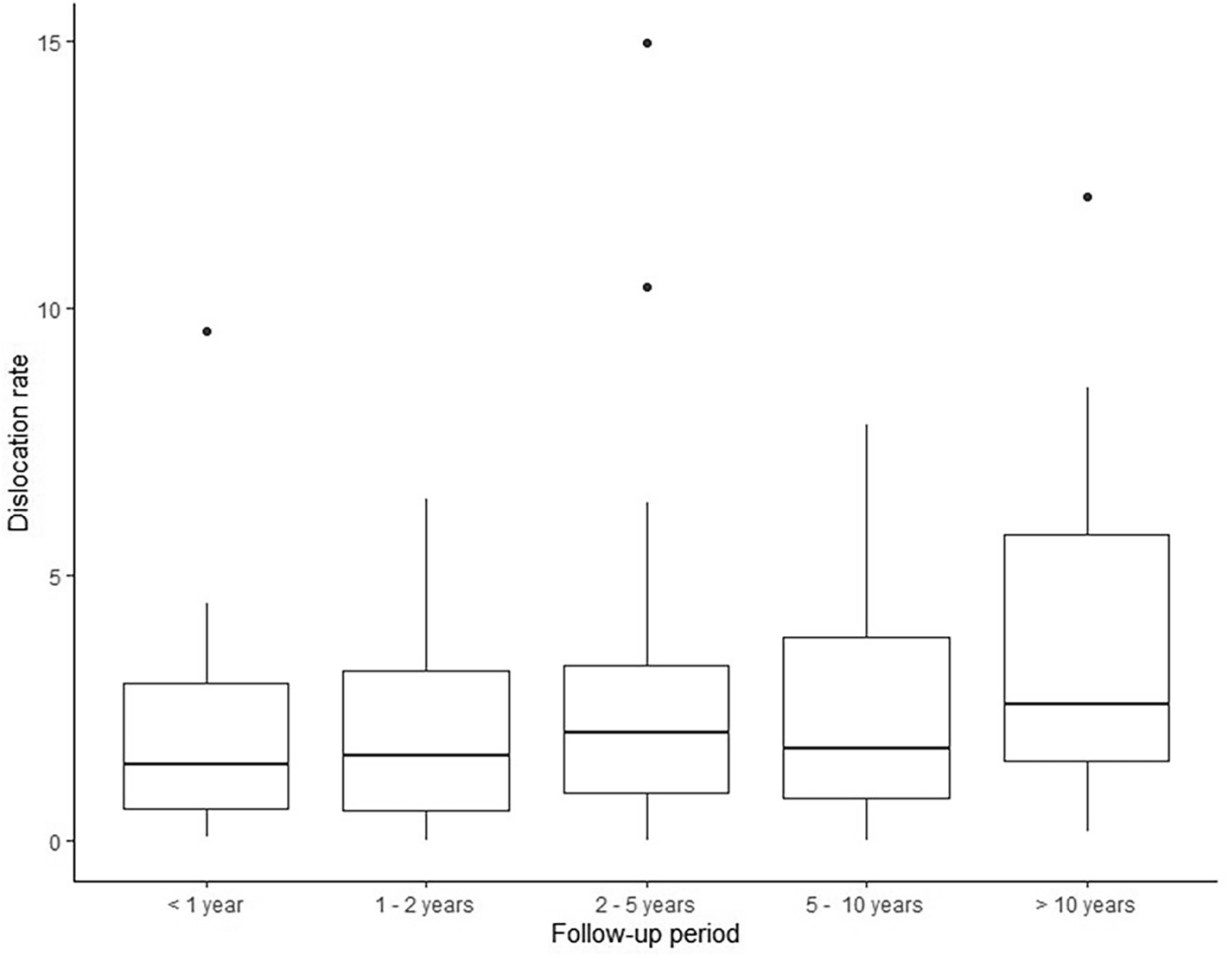
Dislocation rate (%) per length of follow up

**Fig. 4:**
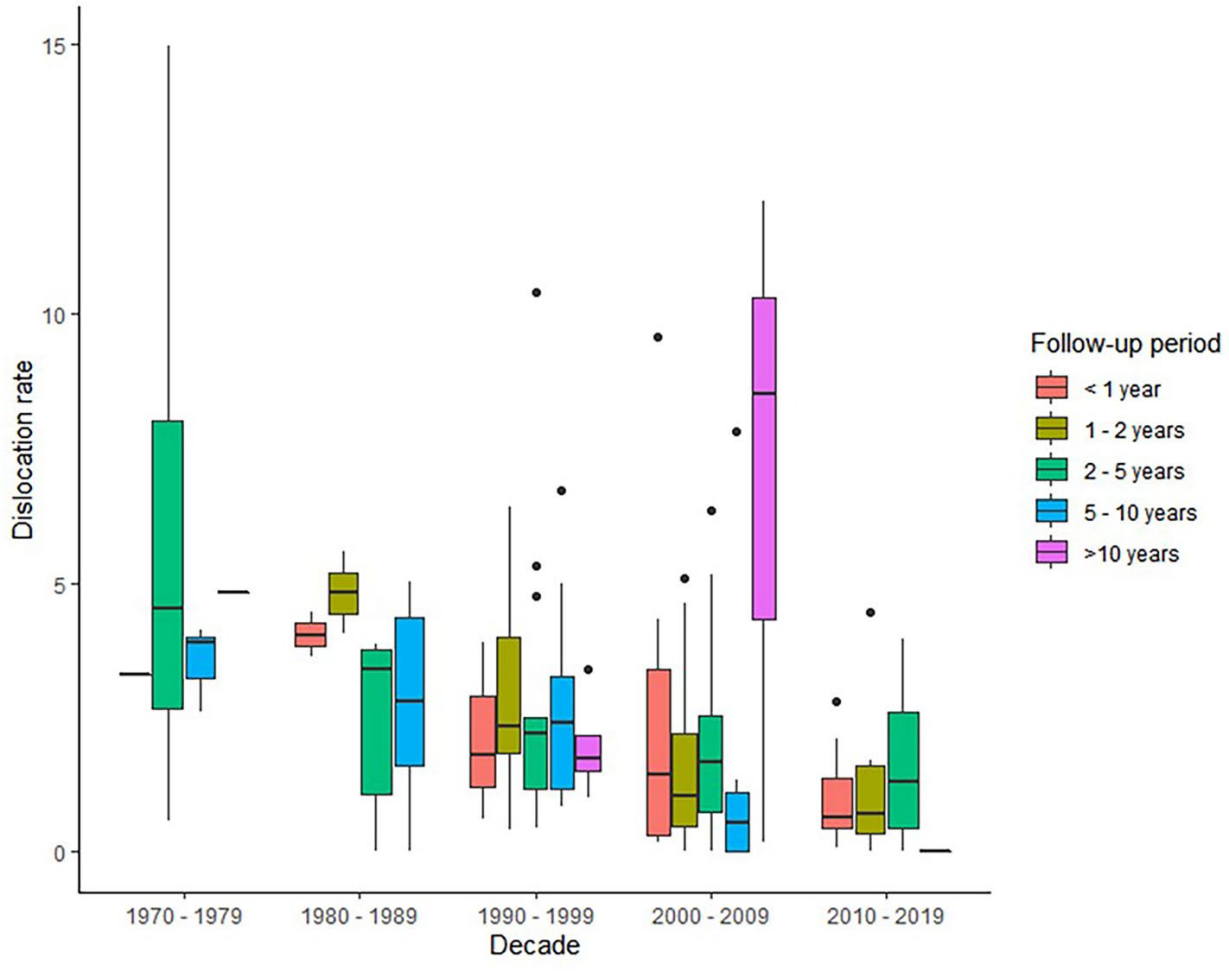
Dislocation risk per decade subdivided by follow-up period

In Fig. 3, the dislocation risk is shown according to follow-up duration, and in Fig. 4, this is subdivided for each decade. The overall dislocation risk was higher when the follow-up duration was longer. However, this difference was not statistically significant (*p* = 0.903). Table 3 shows the overall risk and yearly dislocation rate for different study designs, decades and studies with and without RWD. In studies with RWD, the overall risk and yearly rate were 2.1 and 1.0%, respectively, whereas those were 1.4 and 1.1% in studies without RWD.

**Table 3:** Dislocation rate stratified by study design are shown

Study type	Number of studies (all/with > 1 yr FU)	Median FU in months	*n* THA	*n* dislocations	Overall dislocation risk (%)	Yearly dislocation rate* (%)
Total	174/139	24	5.030.293	85.209	1.7	1.0
Retrospective cohort	91/79	27	333.148	5.552	1.7	1.0
Prospective cohort	46/38	24	76.272	1.972	2.6	0.9
Registry	32/18	12	4.619.312	77.641	1.7	1.1
RCT	5/4	12	1.561	44	2.8	2.1
Studies with RWD	55/55	31	1.920.102	40.217	2.1	1.0
Studies without RWD	119/85	23	3.110.191	44.992	1.4	1.1

*FU* follow-up, *RWD* real-world data, as defined as a minimum of 1000 patients and 1 year FU

*Yearly dislocation rates are based on data from studies with a minimum of 1 year follow-up

Table 4 shows the multivariate meta-regression analysis for follow-up period, sample size, year in which the study started and study design. While study design was an independent predictor for the dislocation risk, length of follow-up and sample size were not (*p* = 0.903 and *p* = 0.755, respectively). Registry studies reported significantly higher dislocation rates (estimate 0.573; 95% CI 0.087–1.060; *p* = 0.021) compared to other designs after adjusting for follow-up period, sample size and year in which the study started.

**Table 4:** Multivariate meta-regression analysis

	Estimate (CI)	SE	*Z* value	*p* value
Follow-up length	< 0.001 (− 0.005, − 0.004)	0.002	− 0.112	0.903
Study year	− 0.047 (− 0.062, − 0.032)	0.008	− 5.976	< 0.001
Total number of THAs	< 0.001(− < 0.001, − < 0.001)	< 0.001	− 0.312	0.755
Type of study				
Prospective cohort (reference)	0 (NA)	NA	NA	NA
RCT	0.388 (− 0.564, − 1.340)	0.485	0.797	0.425
Retrospective cohort	0.075 (− 0.301, − 0.045)	0.192	0.391	0.696
Registry	0.573 (0.087, − 1.060)	0.248	2.311	0.021

Estimated differences of factors affecting dislocation rate

*THA* total hip arthroplasty, *CI* confidence interval, *SE* standard error, *NA* not applicable, *RCT* randomized control trial

## Discussion

The aim of this meta-analysis was a thorough analysis of the evolution of the dislocations risk of primary THAs over the past decades and evaluation of its potential publication bias. Over the last 50 years, the overall dislocation risk was 1.7%; however, it declined from 3.7% in the 1970s to 0.7% the last decade. Analysis of the yearly dislocation rates demonstrated a decrease in improvement over the decades. Investigators from the Mayo clinic described a cumulative dislocation risk in a series of more than 6000 THAs of 1.9% after 1 month and 1.8% at 1 year, which are comparable to our results. The risk rose at a constant 1% for every additional 5 years of follow-up, reaching 7% at 20 years. The risks were greater in older patients (> 70 years old) and in females [25].

The decrease in dislocation risk could be a result of improvement in surgical techniques, such as muscle sparing, alternative approaches, increased head sizes and improved implant design [26, 27]. Optimized implant positioning with restauration of femoral offset contributes to THA stability as well [28, 29]. Recent studies have confirmed the importance of capsular repair; optimal stability is acquired with transosseous bone sutures, instead of capsular resection [30, 31]. No difference was found between absorbable and non-absorbable sutures for this repair [32]. Improved recognition by surgeons of patient-related risk factors for dislocation might also be contributing.

However, patient-related risk factors, as well as patient selection have changed over time [33, 34]. Therefore, it can be argued, that improvements in dislocation rates are not the result of technical and surgical improvements alone. Performing surgery on more females with extremes of age, with a higher BMI or patients with a higher level of frailty or spinal stiffness, without a long-term follow-up might miss out on late dislocations [4, 13, 26, 33–37]. Broadening of the indication for THA such as THA placement for pathological fractures or acetabular or femoral neck fractures might contribute as well [38–40]. This causes an overall decline in dislocation rate, but a stable yearly dislocation rate.

Reported complication rates are also influenced by the quality of reporting in the THA literature. Our analysis shows that no decrease of dislocation rates have been made since the 1980s. Additionally, due to a time-lag bias in limited follow-up of studies performed in the latest decade, the dislocation rate might actually be increasing. The decline in follow-up over the decades combined with a change in patient population could have attributed to this.

Furthermore, despite the improved methods for registration of dislocations, such as digitalization, (national) implant registries and international scientific society databases, we believe that the reported risk and rate of dislocation is still an underestimation of the true prevalence and incidence. As a result of attrition bias, thus patients tending to move, migrate or being transferred to other hospitals, not all dislocations will be reported. This is confirmed by the finding that the reported dislocation risk is not related to the length of follow-up in the meta-regression analysis. This can be explained by a median follow-up that was relatively short (24 months) in all studies, with a low proportion of studies with > 10-year follow-up. The high dislocation risk in studies with > 10-year follow-up in the 2000s (Fig. 3) demonstrates the reporting bias introduced when long-term dislocations are not included. It can be expected that early dislocations are often registered as complications, while late dislocations might not be registered. Late dislocations are often caused by implant wear and/or by patient-specific changes, such as alterations in spinopelvic alignment or soft tissue atrophy, which can become clinically relevant years after THA implantation [11, 12].

Devane et al. retrospectively studied the true dislocation rates of their hospital compared to the rates in the national registry of New Zealand [41]. They found that revisions were recorded correctly, but patients who were treated with a closed reduction only (57%), were often not identified. Similarly, a Danish study from Hermanssen et al. also showed a lower registration of dislocations in the national register compared to the true rate, and only 50% of the true incidence was identified [42]. Since part of the dislocations remain stable after the first dislocation, the true dislocation rate will be underestimated [3, 22, 23]. For this reason, in this review only registry studies that reported all dislocations, and not only revisions, were included. In a large registry study by Bozic et al. a 3.7% revision rate due to instability (48.217 revisions due to instability in 1.300.666 THAs) was reported [43]. To determine the true dislocation risk in THA, registries should also include dislocations for which no revision surgery was performed. Over time evolving patient-, surgery- and implant-related confounders should be included as well and should be the focus of future research, as part of the post-market surveillance. This might lead to continuous monitoring of the real dislocation rate and can be used to improve clinical outcomes and patient information.

The guidelines of many orthopedic journals classify RCTs as the highest level of evidence. RCTs are designed to avoid bias, but are not necessarily the best design for collection of epidemiological data on surgical complications. Even when randomization and blinding are successful, and crossover of patients is adequately dealt with, the results will often be limited to a selected subgroup and depending on surgical experience of selected surgeons. In 1996, Black et al. discussed the RCT’ low external validity: RCTs are designed to test for efficacy, but are only representative for very specific patients, because of the strictly controlled test population [44]. RWD can only be obtained by very large observational studies and registries with sufficient follow-up [44–46]. Registry studies also have disadvantages: a limited availability of patient data and follow-up, no control population and possible duplications or wrong inclusion of patients because of anonymity and miscoding. However, a meta-analysis of Abraham (2010) showed that results of well-designed non-randomized studies, including registries, are just as accurate as that from RCTs [47]. Furthermore, in general, RCTs and smaller cohort studies are often contract research, as compared to investigator or society initiated research in large-scale cohorts. In our study was the reported dislocation risk higher in the large-scale cohorts and registry studies (RWD), compared to the other studies. The reported yearly dislocation rates, however, were comparable between studies with and without RWD.

The data from this thorough meta-analysis show that, for the purpose of reflecting the actual dislocation risk, data from large-scale cohorts and registries are superior to the data obtained from medium-size RCTs. It is likely that the higher dislocation risk reported in studies with RWD is more accurate than the non-RWD and underreporting is the general weakness. Therefore, these non-randomized large-scale cohorts and registries should be considered the highest level of evidence in this type of surgical research.

Because of the enormous heterogeneity and lack of reporting in many studies, confounders such as patient-related factors, for example body mass index and American Society of Anaesthesiologists score, population characteristics, surgical technique and approach and implants used, were not taken into account. This can be considered a limitation of this review, and no conclusions on the effect of individual technical improvements on the dislocation risk can be drawn. In this review, cohort studies or RCTs with relatively small sample size (*n* < 100) were not included, because of the risk of selection bias and the low incidence of dislocations.

## Conclusion

The overall dislocation risk is 1.7% after primary THA. The overall dislocation risk in THA has been decreasing from 3.7 to 0.7%, with no improvement in the yearly dislocation rate. We found no significant reporting bias on the dislocation rates regarding sample size and follow-up duration. Articles with registry data reported a higher dislocation risk compared to other study types. This indicates underreporting of dislocations in RCTs and smaller cohort studies. Therefore, large-scale cohorts and registries should be seen as a more accurate representation of real-world data.

## Supplementary information

Below is the link to the electronic supplementary material.

Supplementary 1 (DOCX 134 KB)

## Data Availability

Not applicable.
