# Supplementary material for: Did the dislocation risk after primary total hip arthroplasty decrease over time? A meta-analysis across six decades
Source: Arch Orthop Trauma Surg. 2022 Nov 10;143(7):4491–500. doi: 10.1007/s00402-022-04678-w (PMC10293125; doi:10.1007/s00402-022-04678-w)
Supplement: Supplementary file 1 — Supplementary 1 (DOCX 134 KB) [file 402_2022_4678_MOESM1_ESM.docx]

**Supplementary Data**

**Figure 1: Funnel plot of percentage of dislocation per study.**


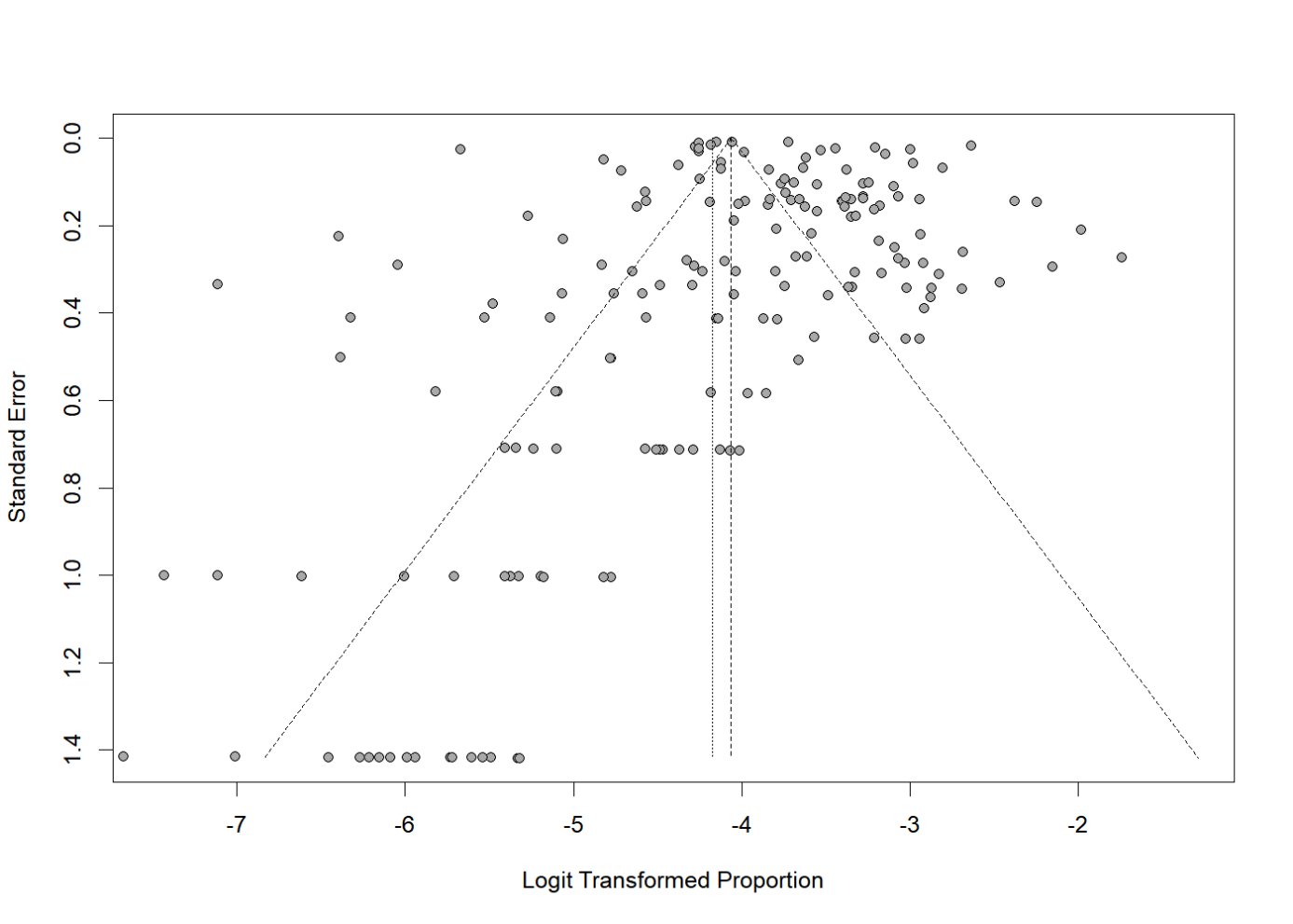


**Table 1. Search syntaxes.**

**PubMed**

| Search | Query | Items found |
| --- | --- | --- |
| #1 | Search **(((((((Hip replacement surgery[Title/Abstract]) OR Hip Joint replacement[Title/Abstract]) OR Hip arthroplasty[Title/Abstract]) OR Total hip arthroplasty[Title/Abstract]) OR THA[Title/Abstract]) OR Total hip replacement[Title/Abstract]) OR Hip prosthesis[Title/Abstract]) OR Total Hip prosthesis[Title/Abstract]** | [31532](https://www.ncbi.nlm.nih.gov/pubmed/?cmd=HistorySearch&querykey=1) |
| #2 | Search **(((Hip joint dislocation[Title/Abstract]) OR Dislocation[Title/Abstract]) OR Hip joint luxation[Title/Abstract]) OR Luxation[Title/Abstract]** | [443](https://www.ncbi.nlm.nih.gov/pubmed/?cmd=HistorySearch&querykey=2)58 |
| #3 | Search **((((primary surgery[Title/Abstract]) OR Primary[Title/Abstract]) OR (First surgery[Title/Abstract])))** | 1395004 |
| #4 | Search **(#1 AND #2 AND #3)** | 989 |

**Embase**

| Search | Query | Items found |
| --- | --- | --- |
| #1 | **'hip replacement surgery'**:ti,ab,kw OR **'hip joint replacement'**:ti,ab,kw OR **'hip arthroplasty'**:ti,ab,kw OR **'total hip arthroplasty'**:ti,ab,kw OR **'tha'**:ti,ab,kw OR **'total hip replacement'**:ti,ab,kw OR **'hip prosthesis'**:ti,ab,kw OR **'total hip prosthesis'**:ti,ab,kw | [40,2](https://www.embase.com/)83 |
| #2 | **'hip joint dislocation'**:ti,ab,kw OR **'dislocation'**:ti,ab,kw OR **'hip joint luxation'**:ti,ab,kw OR **'luxation'**:ti,ab,kw | [49,3](https://www.embase.com/)83 |
| #3 | **'primary surgery'**:ti,ab,kw OR **'primary'**:ti,ab,kw OR **'first surgery'**:ti,ab,kw | 1,922,887 |
| #4 | #1 AND #2 AND #3 | 1,254 |

**Cochrane**

| Search | Query | Items found |
| --- | --- | --- |
| #1 | (Hip replacement surgery):ti,ab,kw OR (Hip Joint replacement):ti,ab,kw OR (Hip arthroplasty):ti,ab,kw OR (Total hip arthroplasty):ti,ab,kw OR (THA):ti,ab,kw OR (Total hip replacement):ti,ab,kw OR (Hip prosthesis):ti,ab,kw OR (Total Hip prosthesis):ti,ab,kw | [573](https://www.ncbi.nlm.nih.gov/pubmed/?cmd=HistorySearch&querykey=1)5 |
| #2 | (Hip joint dislocation):ti,ab,kw OR (Dislocation):ti,ab,kw OR (Hip joint luxation):ti,ab,kw OR (Luxation):ti,ab,kw | 1411 |
| #3 | (primary surgery):ti,ab,kw OR (Primary):ti,ab,kw OR (First surgery):ti,ab,kw | 193165 |
| #4 | Search **(#1 AND #2 AND #3)** | [139](https://www.ncbi.nlm.nih.gov/pubmed/?cmd=HistorySearch&querykey=6) |

**Removing Duplicates via Endnote**

- 1128 articles

**Meeting inclusion/exclusion criteria**

- 170 articles

**Table 2. Inclusion process**

| **Author** | **Publication year** | **Inclusion of consecutive patients** | **Representativeness (multicenter)** | **> 80% follow-up** | **Minimization of potential confounding** |
| --- | --- | --- | --- | --- | --- |
| Abdel, M. P. | 2016 | - | - | + | - |
| Ali, M. S. | 2003 | - |  | + | - |
| Allen, F. C. | 2019 | - | - |  | - |
| Amando, O. | 2018 | + |  | + | - |
| Annan, J. | 2018 | + | - | + | + |
| Attenello, J. | 2019 | - | - |  | + |
| Barkatali, B. M. | 2018 | - | - | + | - |
| Barnett, S. L. | 2016 | + | + | + | - |
| Berend, K. R. | 2017 | + | - | - | - |
| Berry, D. J. | 2005 | + | - |  | + |
| Berry, D. J. | 2004 | + | - |  | + |
| Bhandari, M. | 2009 | + | + |  | + |
| Blakeney, W. G. | 2018 | - |  | + | - |
| Blom, A. W. | 2008 | + | - | + | - |
| Bohl, D. D. | 2019 | + |  |  | + |
| Bono, O. J. | 2018 | + | - |  | - |
| Bouchet, R. | 2011 | + | - | + | - |
| Bozic, K. J. | 2010 | - |  | + | + |
| Brennan, S.A. | 2012 |  | - |  | - |
| Brien, W. W. | 1993 | + | - |  | - |
| Browne, J. A. | 2012 | + | - | + | - |
| Buckland, A. J. | 2017 |  |  |  | - |
| Cafri, G. | 2017 | - |  |  | + |
| Callaghan, J. J. | 2001 |  | - |  | + |
| Castellini, I | 2016 | - |  | - | - |
| Caton, J. H. | 2014 | - | - | + | - |
| Chen, M. | 2017 | - | - | + | - |
| Chiu, F. Y. | 2000 |  |  | + | + |
| Choy, W. S. | 2013 | + | - | + | - |
| Cobb, T.K. | 1996 |  | - |  | - |
| Cullen, C. | 2006 | + | + |  | - |
| Curtin, P. | 2011 | - | + | + | - |
| Danoff, J. R. | 2016 |  | - | + | + |
| Davis, A.M. | 2011 | - | - | - | + |
| Dayican, A. | 2004 | + | - | + | - |
| de Palma, L. | 2012 | + | - |  | - |
| Delaunay, C. | 2014 | - | - | + |  |
| Demos, H. A. | 2001 | - |  |  | - |
| Devane, P.A. | 2012 |  | + | + | - |
| Dixon, M.C. | 2004 |  | - | + | - |
| Edmunds, C.T. | 2011 | - | + |  | - |
| Fender, D. | 1999 | - | + | + | - |
| Fuijshiro, T. | 2016 | + | - | + | + |
| Fuijta, H. | 2012 | - | + |  | - |
| Gausden, E.B | 2018 |  | + |  | + |
| Ghanem, M. | 2013 |  | - |  | - |
| Goel, A. | 2015 | - | + |  | - |
| Gofton, W. | 2015 | - | + |  | - |
| Gofton, W. | 2017 |  | - | + | - |
| Goldberg, V.M. | 1996 | + | - | + | - |
| Grammatopoulos, G. | 2015 | - | + | - | + |
| Grano, G.F. | 2016 |  | - | + | - |
| Grant, J.A. | 2018 | - | - | + | + |
| Gray, A. | 2005 | - | + |  | + |
| Gromov, K. | 2015 | + | - | + | - |
| Gulati, A. | 2008 | + | - | + | - |
| Hasegawa, Y | 2015 |  | - | + | - |
| Haughom, B.D. | 2016 |  | - | + | - |
| Hedlundh, U. | 1996 |  | + | - | + |
| Ho, K.W. | 2012 | + |  | + |  |
| Homma, Y. | 2016 | + |  | + | - |
| Howie, D.W. | 2012 | - |  |  | - |
| Husted, H. | 2010 | + | + |  | - |
| Illgen, R.L | 2017 | + | - |  |  |
| Iorio, R. | 2006 | + | - | + | - |
| Jameson, S.S. | 2011 | - | + |  | - |
| Johnson, R.L. | 2019 | + | - |  | + |
| Kaneko, T. | 2014 | - | + |  | + |
| Katz, J.N. | 2001 | - | + |  | + |
| Kelley, S.S. | 1998 | - | - | + | - |
| Khatod, M. | 2006 | - | + |  | + |
| Kim, H.J. | 2018 | + | - | + | - |
| Kim, M.W. | 2017 | - | - | + | - |
| Kim, S.C. | 2017 | - | - | + | - |
| Kim, Y.H. | 2009 | + |  |  | + |
| Kim, Y.S. | 2008 |  | - | + | - |
| Kornuijt, A. | 2016 | + | - | + | + |
| Krenzel, B.A. | 2010 | - | + |  | - |
| Kurtz, S.M. | 2017 | - | + |  | + |
| Kurtz, S.M. | 2016 | - |  |  | - |
| Lawton, R.L | 2004 | - | - |  | - |
| Lee, D.W. | 2014 | - |  | + | - |
| Lee, S.W. | 2016 | + | - | + | - |
| Legenstein, R. | 2016 |  | - | - | - |
| Li, E. | 1999 | + |  |  | - |
| Lim, S.J. | 2018 |  | - | + | - |
| Lindberg, H.O. | 1982 |  |  |  | - |
| Lombardi, A.V. | 2011 |  |  | + | - |
| Lubbeke, A. | 2009 | + | - | + | + |
| Luzzi, A.J. | 2018 | + |  |  | - |
| Maempel, J.F. | 2016 | - | - | + | + |
| Mahomed, N.N. | 2003 | - | + |  | + |
| Malek, I.A. | 2016 | + | - | + | - |
| Malkani, A.L. | 2010 |  | + |  | + |
| Malkani, A.L. | 2017 |  | + |  | + |
| Mallory, T.H. | 1999 | + | - | + | - |
| Matta, J.M. | 2005 | + | - | + | - |
| Meding, J.B. | 1999 | + |  |  | - |
| Mirza, A.J. | 2014 | + | - | + | - |
| Moran, M. | 2005 |  |  |  | + |
| Moskal, J.T. | 1996 | + | - |  |  |
| Nahas, S. | 2018 |  | - | - | - |
| Nakai, T. | 2014 | + | - | + | - |
| Nakashima, Y. | 2014 | - | - | + | + |
| Newington, D.P. | 1990 | + | - |  | - |
| Norambuena, G.A. | 2019 | + | - | + | - |
| Pai, V.S. | 1997 | - | - | + | - |
| Palan, J. | 2009 |  | - | - | - |
| Paterno, S.A | 1997 | + | - | + | + |
| Paxton, E.W. | 2015 |  | + | + | + |
| Peak, E.L. | 2005 | - | - | + | + |
| Peter, R. | 2011 | + | - | + | + |
| Peters, C.L. | 2007 |  | + | + | - |
| Phillips, C.B. | 2003 |  | + | + | - |
| Prudhon, J.L. | 2017 | - |  | + | - |
| Ravi, B. | 2014 | - |  | - | + |
| Restrepo, C. | 2011 | + | - | + | - |
| Retpen, J.B. | 1989 | + | - | - | - |
| Ricciardi, B.F. | 2017 | + | - |  | + |
| Rinaldi, G. | 2018 | + | - | + | - |
| Robinson, M. | 2012 | + | - | + | - |
| Rowan, F.E. | 2017 |  |  |  | - |
| Sadr Azodi, O. | 2008 |  | + |  | + |
| Saleh, A. | 2019 | + | + | - | + |
| Sanchez-Sotelo, J. | 2006 | + | - |  | - |
| Sandiford, N.A. | 2014 |  | - |  | - |
| Sariali, E. | 2017 | + | - | + | - |
| Schmidt-Braekling, T. | 2015 | + | - | + | - |
| Seagrave, K. | 2017 | + | + | + | + |
| Sheth, D. | 2015 |  | + | + | + |
| Sierra, R.J. | 2005 |  | - | + | - |
| Sing, D.C | 2016 |  | + |  | - |
| Singh, S.P. | 2013 | + | - | + | - |
| Sioen, W. | 2017 | + | - | + | - |
| Smit, M.J | 2009 | + | - | + | - |
| Soballe, K. | 1987 | + | - | + | - |
| Solomon, D.H. | 2002 |  | + |  | + |
| Soohoo, N.F. | 2010 |  | + |  | + |
| Spaans | 2015 | + | - | + | - |
| Suh, K.T. | 2004 | + | - |  | - |
| Surace, M.F. | 2016 | + | - |  | + |
| Talbot, N.J. | 2002 | + |  | + | - |
| Tarasevicius, S. | 2017 |  | + |  | + |
| Tian, S. | 2019 | + | - |  | - |
| Timperley | 2016 | + | - |  | + |
| Tsai, S.J. | 2008 | + | - | + | - |
| Tsai, S.W. | 2015 | + | - | + | + |
| van der Weegen, W. | 2019 | + |  | + | + |
| van Stralen | 2003 | + | - | + | - |
| Vicar, A.J. | 1984 |  |  | + |  |
| Vicente, J.R. | 2009 | + | - |  | - |
| von Knoch, M. | 2002 |  | - |  | - |
| Wagner, E.R. | , 2016 | + | - |  | + |
| Wang, W. | 2014 | + |  | + | - |
| Weeden, S.H. | 2003 |  |  |  | - |
| Werner, B.C. | 2017 |  | + | + | + |
| White, R.E. | 2001 | + | - |  | - |
| Williams, J.F. | 1982 | + |  |  | - |
| Williams, O. | 2002 |  | + |  | - |
| Wittenberg, R.H. | 2013 | - | + | + | - |
| Woolson, S.T. | 1999 |  | - | + | - |
| Yang, W.E. | 1998 |  | - |  | + |
| Yoon, J.S. | 2017 |  | - | + | - |
| York, P.J. | 2018 |  |  | + | - |
| Yoshii, H. | 2016 | - | - | - | - |
| Yuan, L. | 1999 | + |  |  | + |
| Zhang, Y. | 2013 | + | - | + | - |
| Zhang, Z.J | 2012 | + | - | + | - |
| Zimmerma, S. | 2002 | - | + | + | + |
|  |  | **46%** | **23%** | **53%** | **31%** |
